# Path-Planning System for Radioisotope Identification Devices Using 4π Gamma Imaging Based on Random Forest Analysis

**DOI:** 10.3390/s22124325

**Published:** 2022-06-07

**Authors:** Hideki Tomita, Shintaro Hara, Atsushi Mukai, Keita Yamagishi, Hidetake Ebi, Kenji Shimazoe, Yusuke Tamura, Hanwool Woo, Hiroyuki Takahashi, Hajime Asama, Fumihiko Ishida, Eiji Takada, Jun Kawarabayashi, Kosuke Tanabe, Kei Kamada

**Affiliations:** 1 Department of Energy Engineering, Nagoya University, Nagoya 464-8603, Japan; hara.shintaro@e.mbox.nagoya-u.ac.jp (S.H.); mukai.atsushi.k5@s.mail.nagoya-u.ac.jp (A.M.); yamagishi.keita@f.mbox.nagoya-u.ac.jp (K.Y.); ebi.hidetake.g5@s.mail.nagoya-u.ac.jp (H.E.); 2 Department of Nuclear Engineering and Management, The University of Tokyo, Tokyo 113-8656, Japan; shimazoe@bioeng.t.u-tokyo.ac.jp (K.S.); leo@n.t.u-tokyo.ac.jp (H.T.); 3 Department of Robotics, Tohoku University, Sendai 980-8579, Japan; ytamura@tohoku.ac.jp; 4 Department of Mechanical Systems Engineering, Kogakuin University, Hachioji 192-0015, Japan; at13710@g.kogakuin.jp; 5 Department of Precision Engineering, The University of Tokyo, Tokyo 113-8656, Japan; asama@robot.t.u-tokyo.ac.jp; 6 National Institute of Technology, Toyama College, Toyama-shi 939-8630, Japan; ishida-f@nc-toyama.ac.jp (F.I.); takada@nc-toyama.ac.jp (E.T.); 7 Department of Nuclear Safety Engineering, Tokyo City University, Tokyo 158-8557, Japan; jkawara@tcu.ac.jp; 8 National Research Institute of Police Science, Chiba 277-0882, Japan; tanabe@nrips.go.jp; 9 Institute of Engineering Innovation, Tohoku University, Sendai 980-8579, Japan; kamada@imr.tohoku.ac.jp

**Keywords:** path planning, radioisotope identification, 4π gamma imaging, random forest

## Abstract

We developed a path-planning system for radiation source identification devices using 4π gamma imaging. The estimated source location and activity were calculated by an integrated simulation model by using 4π gamma images at multiple measurement positions. Using these calculated values, a prediction model to estimate the probability of identification at the next measurement position was created by via random forest analysis. The path-planning system based on the prediction model was verified by integrated simulation and experiment for a ^137^Cs point source. The results showed that ^137^Cs point sources were identified using the few measurement positions suggested by the path-planning system.

## 1. Introduction

Radiation sources should be handled carefully and controlled strictly. However, in the events of theft and loss of sources or undesired acts of terrorism using such sources, it is necessary to identify multiple sources over a wide search area rapidly [1]. Several methods for radiation source identification have been developed [2,3,4,5,6,7]. For example, Huo et al. reported a method to estimate the location and intensity of radiation sources by using a mobile robot equipped with a Geiger–Müller (GM) counter and laser range sensor [4]. They investigated the selection of the measurement position by reinforcement learning for the autonomous identification of the radiation sources. Besides this, methods to visualize the gamma-ray intensity in an environmental three-dimensional map were developed. Vetter et al. demonstrated radiation source detection using both simultaneous localization and mapping (SLAM) based on a light detection and ranging (LiDAR) system and gamma-ray images obtained using gamma imaging methods such as coded-aperture and Compton imaging [5]. Sato et al. applied this method to visualize the gamma-ray intensity at the site of Fukushima Daiichi Nuclear Power Plant [6,7].

The typical field of view of conventional gamma imaging is less than 180°, whereas 4π Compton imaging is sensitive to gamma rays incident on a detector from all directions. Hence, 4π Compton imaging can allow more rapid identification of radiation sources than that possible by methods based on conventional gamma imaging. In this regard, we developed a 4π gamma-ray imaging system using gadolinium aluminum gallium garnet (GAGG) scintillators [8,9] and CdTe detectors [10,11]. Previous studies demonstrated that the location and activity of hidden gamma-ray sources can be estimated by combining gamma-ray images measured at multiple positions [11,12]. In autonomous source identification by a 4π Compton imager mounted on a robotic vehicle, it is necessary to optimize the measurement procedure, i.e., the measurement positions around the target sources. For this purpose, we proposed a detector movement algorithm for a single source [13,14]. In this study, we developed a path-planning system for radioisotope identification devices by using 4π gamma imaging based on random forest (RF) analysis.

## 2. Investigation of Path-Planning System Using an Integrated Simulation Model

In the source identification method based on 4π gamma imaging [12], a point source is assumed to exist in a certain voxel in a three-dimensional (3D) voxel space, and the source intensity at the pixel in the direction estimated from the gamma image is calculated from the intensity of the gamma image. This calculation is performed for 4π gamma images obtained at several positions around a source, and finally, the source is identified as being present at that intensity at a position for which the results are consistent. For rapidly identifying radiation sources using 4π gamma imaging, the images should be obtained from multiple positions that are suitable for obtaining the intensity and position information of the sources. In a previous study, we developed a detector movement algorithm for a single radiation source [13]. The first priority in the algorithm is that the detector is moved to the direction with the highest intensity in the 4π gamma image, and the second one is that the detector is moved away from the direction with the highest intensity in the image and toward the direction with the next-higher intensity in the image.

In this study, we investigated a path-planning system for detector movement. An integrated simulation model [13] that estimates the location and intensity of a single gamma source from gamma images at arbitrary positions around the source was used to develop the path-planning system. To obtain gamma images in the simulation, a 3D multipixel array CdTe detector was assumed as a 4π gamma imager. The basic detector response with sufficiently small counting statistics was obtained by measuring ^137^Cs (2 MBq) placed at 100 cm from the center of the detector for 20 min. For any measurement point in the simulation, the gamma image was calculated by rotating the basic response to the direction of a target source and transforming the intensity of the basic response to follow the inverse square law. Therefore, the background variation and uncertainty caused by the counting statistics in calculated gamma images were not considered in the following discussion.

**Figure 1:**
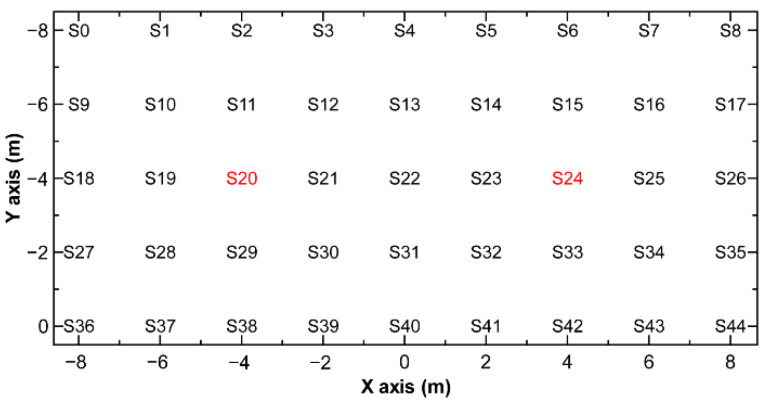
Locations of two-point sources and possible measurement positions around the sources on the search area in the integrated simulation model. The positions of the two sources are indicated in red.

To extract appropriate features in RF analysis even when there are two sources and create a prediction model to estimate the probability of identification at the next measurement position, simulations were performed for two sources. Figure 1 shows the locations of two ^137^Cs point sources and the possible measurement positions around the sources on the search area in the integrated simulation model. The point sources and the possible measurement positions were assumed to be on the same plane. The search area was 8 ≤ X ≤ 8 m and −8 ≤ Y ≤ 0 m. Measurement points A and B were selected from S0 to S44 positions on a 2 m grid in the search area, excluding the two source positions S20 and S24. The intensity ratio of the two sources ranged from 0.1 to 4.9.

**Figure 2:**
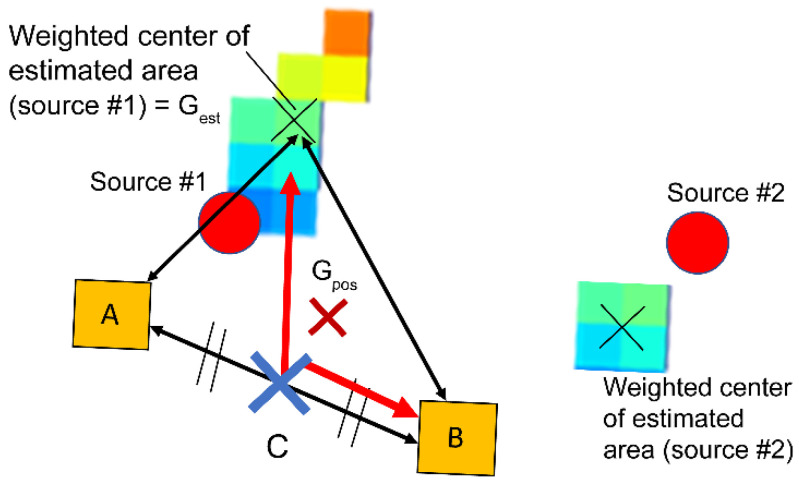
Definition of the candidates of features in the case that the weighted center of the estimated area of source #1 is closer to point C and chosen as the point G_est_.

Simulation of source identification in a 3D voxel space (41 × 41 × 41 voxel, 0.4 m^3^/voxel) was performed under all possible conditions of source intensity ratio and for two measurement positions A and B. The output data were analyzed using RF, a machine learning model. First, to find the features in this RF analysis, the features in the decision tree analysis that were examined in our previous study [13] were selected as the candidates. Figure 2 shows the definitions of the eight candidate features listed below:

$$\mathrm{Line}\;\mathrm{segment}\;\mathrm{between}\;\mathrm{points}\;C\;\mathrm{and}\;G_{\mathrm{est}}\;\left|\vec{{\mathrm{CG}}_{\mathrm{est}}}\right|$$

$$\mathrm{Line}\;\mathrm{segment}\;\mathrm{between}\;\mathrm{points}\;G_{\mathrm{pos}}\;\mathrm{and}\;G_{\mathrm{est}}\;\left|\vec{G_{\mathrm{pos}}G_{\mathrm{est}}}\right|$$

$$\mathrm{Line}\;\mathrm{segment}\;\mathrm{between}\;\mathrm{points}\;A\;\mathrm{and}\;B\;\left|\vec{\mathrm{AB}}\right|$$

$$\mathrm{Line}\;\mathrm{segment}\;\mathrm{between}\;\mathrm{points}\;A\;\mathrm{and}\;G_{\mathrm{est}}\;\left|\vec{{\mathrm{AG}}_{\mathrm{est}}}\right|$$

$$\mathrm{Line}\;\mathrm{segment}\;\mathrm{between}\;\mathrm{points}\;B\;\mathrm{and}\;G_{\mathrm{est}}\;\left|\vec{{\mathrm{BG}}_{\mathrm{est}}}\right|$$

$$\mathrm{Ratio}\;\mathrm{of}\;\left|\vec{{\mathrm{AG}}_{\mathrm{est}}}\right|\;\mathrm{and}\;\left|\vec{{\mathrm{BG}}_{\mathrm{est}}}\right|\;\frac{\left|\vec{{\mathrm{AG}}_{\mathrm{est}}}\right|}{\left|\vec{{\mathrm{BG}}_{\mathrm{est}}}\right|}$$

$$\mathrm{Parallax}\;\mathrm{angle}\;∠{\mathrm{AG}}_{\mathrm{est}}B$$

$$\mathrm{Absolute}\;\mathrm{value}\;\mathrm{of}\;\mathrm{inner}\;\mathrm{product}\;\vec{\mathrm{CB}}\;\mathrm{and}\;\vec{{\mathrm{CG}}_{\mathrm{est}}}\;\left|\vec{\mathrm{CB}}\cdot \vec{{\mathrm{CG}}_{\mathrm{est}}}\right|$$

where A and B are the first and second measurement points, respectively, C is the midpoint of line segment AB, G_est_ is the point closer to point C between the weighted centers of the estimated areas of sources #1 and #2, and G_pos_ is the weighted center of the three points A, B, and G_est_.

Assuming that the source is identified when G_est_ is estimated within ±1 m of the true source location and the estimated source intensity is estimated within ±75% of the true source intensity, the objective function in the RF analysis was set as “detected” (i.e., “1”) or “not detected” (i.e., “0”).

Highly correlated variables, i.e., strong multicollinearity, should be avoided for achieving better accuracy and feature selection in the RF analysis. The variance inflation factor (*VIF*) of the *j*th variable *X_j_*, defined by Equation (1), represents the degree to which one variable is related to other variables, and multicollinearity is suspected if *VIF* is greater than 10.

(1) $$VIF_{j}=\frac{1}{1-R_{j}^{2}}$$

with

(2) $$R_{j}^{2}=1-\frac{\sum_{i=1}^{n}{\left(X_{ji}-f_{ji}\right)}^{2}}{\sum_{i=1}^{n}{\left(X_{ji}-\bar{X_{j}}\right)}^{2}}$$

Here, $R_{j}^{2}$ is the coefficient of determination of the regression equation with *X_j_* on all other remaining variables, $f_{ji}$ is the ordinary least square regression of *X_j_* on the *i*th data, $X_{ji}$ is the *X_j_* value of the *i*th data, $\bar{X_{j}}$ is the mean of *X_j_*, and *n* is the number of data obtained by the simulation. Among the eight variables, $\left|\vec{{\mathrm{CG}}_{\mathrm{est}}}\right|$, $\left|\vec{{\mathrm{BG}}_{\mathrm{est}}}\right|$, and $\left|\vec{G_{\mathrm{pos}}G_{\mathrm{est}}}\right|$ had very large calculated VIFs; hence, these three variables were removed. The results of recalculation of the VIFs for five variables are listed in Table 1. The *VIF* of each feature became smaller (weaker correlation). Since G_est_ is derived from the estimated source identification result, its uncertainty is likely to be larger than the uncertainties of A and B, which can be measured. Therefore, we selected $\left|\vec{\mathrm{AB}}\right|$ and $\left|\vec{\mathrm{CB}}\cdot \vec{{\mathrm{CG}}_{\mathrm{est}}}\right|$ as the features, and calculated the VIFs using each of $\left|\vec{{\mathrm{AG}}_{\mathrm{est}}}\right|$*,* $\frac{\left|\vec{{\mathrm{AG}}_{\mathrm{est}}}\right|}{\left|\vec{{\mathrm{BG}}_{\mathrm{est}}}\right|}$*,* and ∠AG_est_B as an additional variable. The VIF was the lowest when $\frac{\left|\vec{{\mathrm{AG}}_{\mathrm{est}}}\right|}{\left|\vec{{\mathrm{BG}}_{\mathrm{est}}}\right|}$ was included as a feature (see Table 2).

Parameters in the RF analysis were tuned based on grid search and cross-validation. In this analysis, the number of trees was fixed at 50, and the optimal combinations of two parameters, namely, tree depth and the minimum number of data for nodes, were searched for with tree depth set to 1, 2, 3, and 4, and the minimum number of data for nodes set to 1, 3, 5, 7, and 10. After tuning, a model was created with the tree depth and minimum number of data in a node set to 4 and 10, respectively. Finally, a prediction model accuracy of 86% was built. The importance of the features of the extracted decision trees is summarized in Table 3.

**Figure 3:**
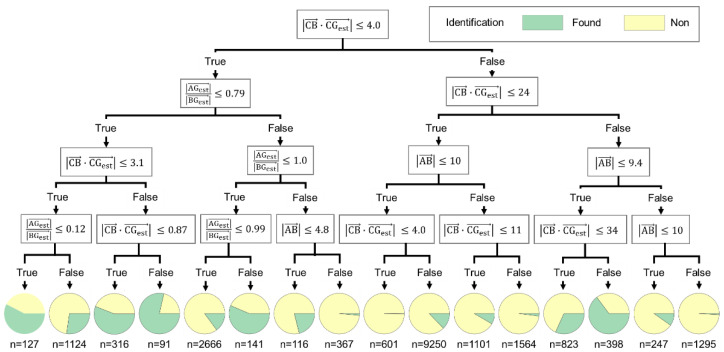
Typical decision trees in this prediction model.

To understand the created prediction model, we extracted typical decision trees with feature importance similar to those of the whole model with 50 decision trees. The output of RF was determined via ensemble learning using multiple decision trees. Here, the python implementation of the CART (classification and regression trees) algorithm for decision trees was used. The model was constructed by recursively partitioning the training data through hierarchical conditional branching. The extracted decision trees are shown in Figure 3. The pie charts in this figure show the percentages of data for which the source was found at the node, and *n* represents the number of data in each node.

On the left node ($\left|\vec{\mathrm{CB}}\cdot \vec{{\mathrm{CG}}_{\mathrm{est}}}\right|\leq 4.0$) in Figure 3, if $\frac{\left|\vec{{\mathrm{AG}}_{\mathrm{est}}}\right|}{\left|\vec{{\mathrm{BG}}_{\mathrm{est}}}\right|}\leq 0.79\;\mathrm{and}\;\left|\vec{\mathrm{CB}}\cdot \vec{{\mathrm{CG}}_{\mathrm{est}}}\right|\leq 3.1$, the possibility of identifying the source is high. This indicates that both the measurement points where the contribution from one source is larger than that from the other and considering the parallax for the source are preferred for selection. This means it is better to find the sources one by one. On the right node ($\left|\vec{\mathrm{CB}}\cdot \vec{{\mathrm{CG}}_{\mathrm{est}}}\right|>4.0$), the possibility is high for $\left|\vec{\mathrm{CB}}\cdot \vec{{\mathrm{CG}}_{\mathrm{est}}}\right|>34,\;\left|\vec{\mathrm{AB}}\right|>9.4$. This is the case when one measurement position is close to the source and the other position is far from the source considering the parallax to the source. Therefore, the measurement positions suggested by the prediction model are also consistent with ones preferred as per the detector movement algorithm for a single source, as discussed in our previous study [13,14].

**Figure 4:**
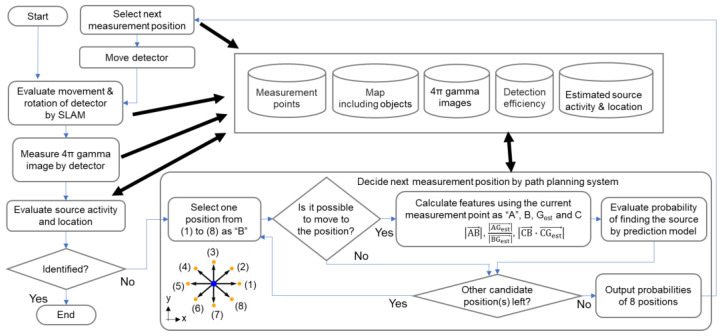
Flowchart of detector movement based on the proposed path-planning system.

We used this prediction model to let the path-planning system decide the next measurement position. Figure 4 shows the flowchart of the proposed path-planning approach to move the detector for identifying radiation sources. After measurement at a certain measurement point A, the location and intensity of the radiation source(s) are estimated according to the identification principle. If the source is not identified with a sufficiently small uncertainty, the path-planning system selects the next measurement point B from eight candidate positions around A. The path-planning system employs the SLAM results to determine whether the detector can move to one of the eight candidate positions. If possible, the probability of identification is estimated by the prediction model using the input features for all the candidates, and the candidate with the highest probability is selected. Then, the detector is moved, and the 4π gamma image is measured. This process is repeated until the source is identified. When measurement locations with the same detection probability were identified by the path-planning system, the next measurement position was selected based on the detector movement algorithm reported in our previous study [13].

## 3. Verification of the Path-Planning System

### 3.1. Verification Based on the Integrated Simulation Model

**Figure 5:**
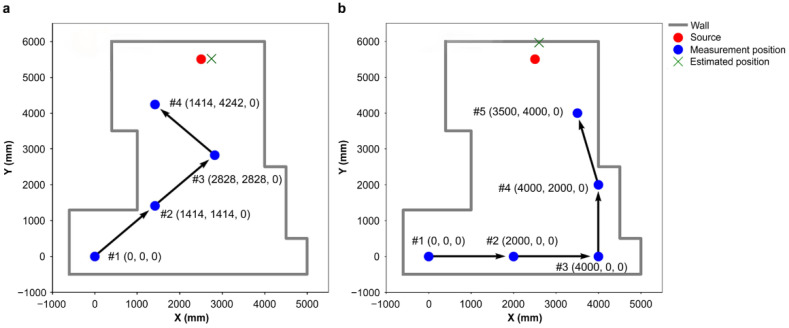
Search area, measurement positions, and movement paths: (**a**) Path I and (**b**) Path II.

As a preparatory step to demonstrate the usefulness of the path-planning system for multiple sources, it was verified whether the system could efficiently identify a single source in this paper. The path-planning system was verified using the integrated simulation model mentioned in Section 2. A ^137^Cs point source (activity: 1.8 MBq) was set within the search area (Figure 5). This search area was designed identically to that in the experiment described in Section 3.2. The source location was at (2.5, 5.5, 0.0), and the initial measurement position was (0, 0, 0) in m. The acquisition time for 4π gamma imaging at each position was 10 min. The distance per step to the next measurement position was 1.5 m. The detector could be moved up to 0.1 m close to the obstacles (i.e., walls) in the search area. The path-planning system was used for selecting the measurement positions. Two patterns of detector movement—Path I selected by the path-planning system, and Path II selected by tracing the edges of the walls in the search area—were investigated, as shown in Figure 5.

**Figure 6:**
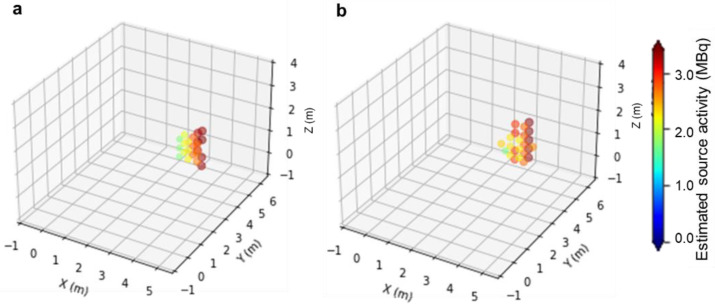
Estimated source intensities and locations in a three-dimensional (3D) voxel space obtained by (**a**) Path I and (**b**) Path II.

Figure 6a shows the estimated source intensities and locations obtained by Path I in a 3D voxel space. The result is summarized in Table 4. In the table, the column “Area where a source is located” indicates how small a source range can be estimated. The column “Estimated source location” gives the weighted centers of the estimated voxels, and “Error from true source position” gives the difference between the true source location and the estimated location. Further, “Estimated source activity” lists the median of the estimated source intensities of all the estimated voxels. As measurements progressed, the estimated source area and the error from the true source location decreased, and in the fourth measurement, the source location could be estimated with an error of less than 0.3 m.

Next, we simulated the source identification using Path II. The results are provided in Table 5 and Figure 6b. Five measurements were required to achieve a source identification accuracy similar to that obtained using Path I. These results show that the detector movement followed by the path-planning system was effective for source identification.

### 3.2. ^137^Cs Source Identification by a Prototype Device by 4π Gamma Imaging with the Path-Planning System

In this study, a prototype 3D multipixel CdTe detector (Hitachi Consumer Electronics Co., Ltd., Hitachi, Japan) was used as a 4π gamma imager. The size of a single CdTe pixel was 8 × 12.5 × 2.2 mm^3^, and the total number of pixels were 8 × 12 × 15 = 1440. These 1440 pixels were arranged in a three-dimensional structure. The energy deposited on each pixel because of the incident gamma rays was recorded as listmode data. The basic performance of the detector as the 4π gamma imager was reported in our previous papers [10,11].

The detector, a 3D-LiDAR (Velodyne Lidar, Inc., San Jose, CA, USA, VLP-16), a spherical camera (Richo, theta-V), a battery, and a laptop PC for acquiring and storing data were mounted on a robotic vehicle (Vstone Co., Ltd., Osaka, Japan, Mecanum Rover ver. 2.1). This device can move for about 4 h without an external power supply. The robotic vehicle can be controlled by a wireless controller and can rotate and move in eight directions. The data from the detector were acquired by the laptop PC and sent together with the 3D-LiDAR data to a control PC for data processing via a wireless connection. In the control PC, SLAM was performed using the 3D-LiDAR data for mapping the surrounding environment and self-position estimation for the vehicle. In addition, the calculations for source identification and decision-making regarding the next measurement position were performed in the control PC.

**Figure 7:**
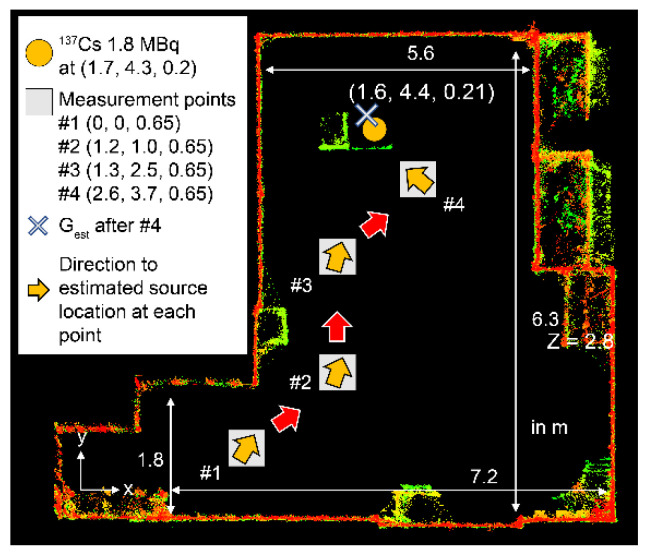
Top view of the room map obtained by simultaneous localization and mapping (SLAM) with the locations of the source and measurement points.

**Figure 8:**
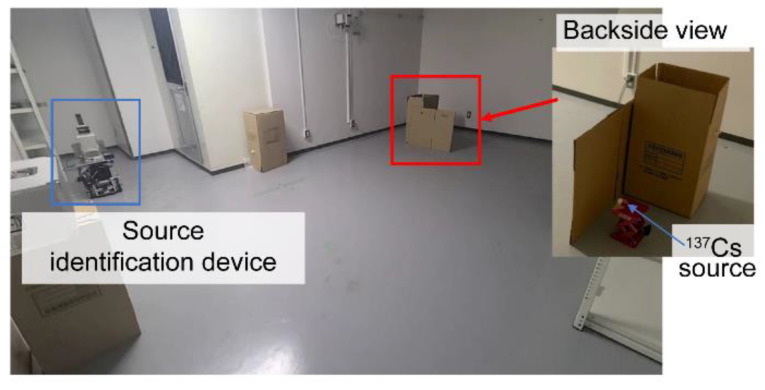
Photograph of the experimental setup.

This experiment was conducted in a room of the same size and under identical conditions as in the simulation (Section 3.1). A top view of the room map with the locations of the source and measurement points is shown in Figure 7. This map was obtained by SLAM with the xy plane at z = 0.2 m. The ^137^Cs point source was placed at (1.7, 4.3, 0.2) m, as shown in Figure 8.

**Figure 9:**
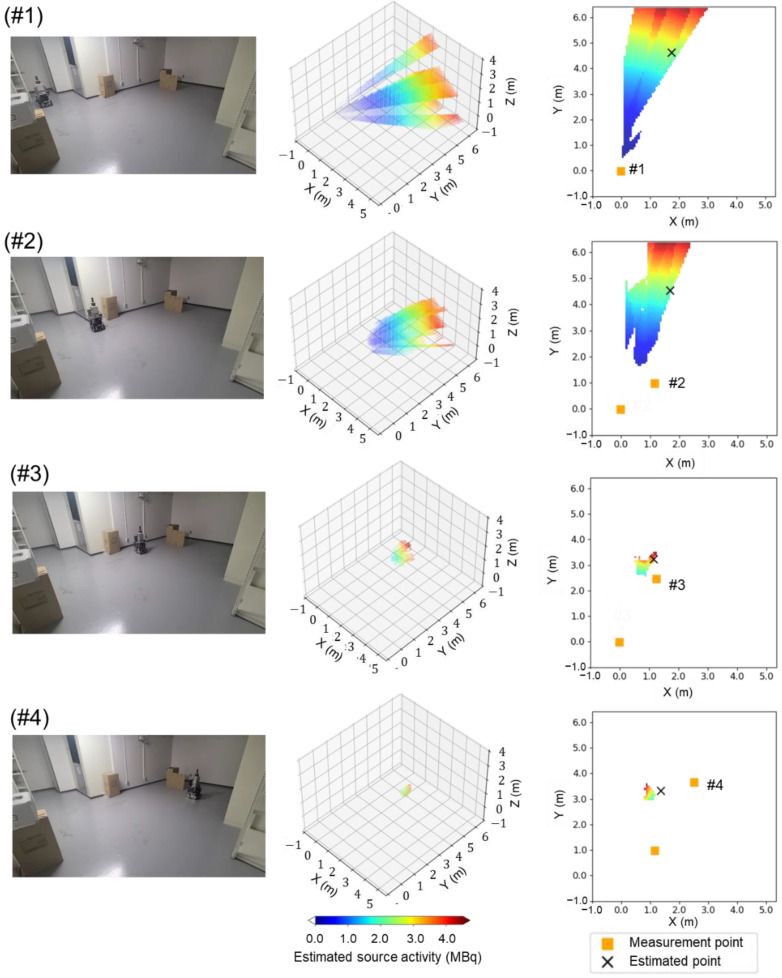
High-angle view of the search area (**left**), estimated source intensity and location in the 3D voxel area (**middle**), and estimation results in the xy plane at z = 0.2 (**right**) for each measurement position.

Table 6 lists the source intensity and location results estimated in this experiment. The top view of the search area is shown in the left column in Figure 9. The identification results of the estimated source intensities and locations in the 3D voxel space and in the xy plane at z = 0.2 obtained as the measurements proceed are also shown in Figure 9. After measurement at the first measurement point (#1), the next measurement position was selected by the path-planning system and the detector was moved. Measurements were taken at the second point. Thus, measurements were taken at four points. After four measurements, the error in the true source position was reduced to ±0.1 m relative to the true source location. The source activity was estimated within a relative error of 40%. Thus, we successfully demonstrated that measurement points #1 to #4 as determined by the path-planning system lead to the estimated source direction autonomously and that the system selects the best measurement positions for ^137^Cs single source identification.

## 4. Conclusions

We developed a path-planning system based on 4π gamma imaging for radiation source identification devices to select measurement positions around locate potential radiation sources. The prediction model was created by performing a RF analysis using the results of an integrated simulation to calculate the estimated source location and activity from 4π gamma images obtained at the measurement points. Using the prediction model, the path-planning system decides the next measurement point. The performance of the path-planning system was verified by a simulation and an experiment using a 4π gamma imager for single ^137^Cs source identification. The simulation and experimental results showed that the ^137^Cs point source was identified with fewer movement steps and high accuracy by using the path-planning system.

Here, the prediction model was based on the results of a simulation model for two sources. The effectiveness of this path-planning system for multiple sources needs to be verified experimentally. For further developing an automated source identification device based on 4π gamma imaging, both the distance to the next measurement point and the measurement time at that point should be optimized in the path-planning system. In addition, the consideration of the effect of shielding surrounding the source is future work.

## Figures and Tables

**Table 1:** Variance inflation factors (VIFs) for five candidate features for radiation source identification.

$\left|\vec{AB}\right|$	$\left|\vec{AG_{est}}\right|$	$\frac{\left|\vec{AG_{est}}\right|}{\left|\vec{BG_{est}}\right|}$	∠AG_est_B	$\left|\vec{CB}\cdot \vec{CG_{est}}\right|$
16	14	15	7.9	3.9

**Table 2:** VIFs for the selected features in the random forest analysis.

$\left|\vec{AB}\right|$	$\frac{\left|\vec{AG_{est}}\right|}{\left|\vec{BG_{est}}\right|}$	$\left|\vec{CB}\cdot \vec{CG_{est}}\right|$
5.3	2.8	2.7

**Table 3:** Importance of the features in the model created by the RF analysis.

	$\left|\vec{AB}\right|$	$\frac{\left|\vec{AG_{est}}\right|}{\left|\vec{BG_{est}}\right|}$	$\left|\vec{CB}\cdot \vec{CG_{est}}\right|$
Model based on all 50 trees	0.36	0.26	0.39
Decision tree extracted from the model	0.35	0.27	0.38

**Table 4:** Summary of the estimated source intensities and locations obtained by Path I in the integrated simulation model.

Movement Sequence	#1	#2	#3	#4
Area where a source is located (voxels)	1208	796	154	22
Estimated source location (m)	(3.8, 5.8, 0.0)	(4.1, 6.3, 0.0)	(3.2, 6.3, 0.1)	(2.8, 5.5, 0.0)
Error from true source position (m)	(1.3, 0.3, 0.0)	(1.6, 0.8, 0.0)	(0.7, 0.8, 0.1)	(0.3, 0, 0)
Estimated source activity (MBq)	3.2 ± 3.2	3.8 ± 3.0	3.5 ± 2.0	2.6 ± 0.9

**Table 5:** Summary of the estimated source intensities and locations obtained by Path II in the integrated simulation model.

Movement Sequence	#1	#2	#3	#4	#5
Area where a source is located (voxels)	1208	1058	746	330	23
Estimated source location (m)	(3.8, 5.8, 0.0)	(3.8, 6.0, 0.0)	(3.1, 6.1, 0.0)	(3.1, 6.5, 0.0)	(2.6, 5.9, 0.0)
Error from true source position (m)	(1.3, 0.3, 0.0)	(1.3, 0.5, 0.0)	(0.6, 0.6, 0.0)	(0.6, 1.0, 0.0)	(0.1, 0.4, 0.0)
Estimated source activity (MBq)	3.2 ± 3.2	3.1 ± 2.6	2.8 ± 1.9	3.2 ± 1.6	2.8 ± 0.8

**Table 6:** Summary of the source intensities and locations estimated in the experiment.

Movement Sequence	#1	#2	#3	#4
Area where a source is located (voxels)	29,551	13,348	592	58
Estimated source location (m)	(2.3, 5.8, 0.6)	(2.2, 5.7, 0.3)	(1.5, 4.1, 0.4)	(2.0, 4.1, 0.4)
Error from true source position (m)	(0.5, 1.5, 0.4)	(0.5, 1.4, 0.1)	(−0.2, −0.2, 0.2)	(0.3, −0.2, 0.2)
Estimated source activity (MBq)	3.1 ± 3.1	3.4 ± 3.1	1.6 ± 1.0	2.1 ± 0.8

## Data Availability

All relevant data used in the evaluation is displayed in the graphs contained within the article. Values for the raw data points are available upon request.
